# Phospholamban overexpression in mice causes a centronuclear myopathy-like phenotype

**DOI:** 10.1242/dmm.020859

**Published:** 2015-08-01

**Authors:** Val A. Fajardo, Eric Bombardier, Elliott McMillan, Khanh Tran, Brennan J. Wadsworth, Daniel Gamu, Andrew Hopf, Chris Vigna, Ian C. Smith, Catherine Bellissimo, Robin N. Michel, Mark A. Tarnopolsky, Joe Quadrilatero, A. Russell Tupling

**Affiliations:** 1 Department of Kinesiology, University of Waterloo, Waterloo, Ontario N2L 3G1, Canada; 2 Department of Exercise Science, Concordia University, Montreal, Quebec H4B 1R6, Canada; 3 Departement of Kinesiology, McMaster University, Hamilton, Ontario L8N 3Z5, Canada; 4 Department of Pediatrics, McMaster University, Hamilton, Ontario L8N 3Z5, Canada; 5 Department of Medicine, McMaster University, Hamilton, Ontario L8N 3Z5, Canada

**Keywords:** SERCA, Dynamin 2, Skeletal muscle, Calcium regulation, Congenital myopathy

## Abstract

Centronuclear myopathy (CNM) is a congenital myopathy that is histopathologically characterized by centrally located nuclei, central aggregation of oxidative activity, and type I fiber predominance and hypotrophy. Here, we obtained commercially available mice overexpressing phospholamban (*Pln*^OE^), a well-known inhibitor of sarco(endo)plasmic reticulum Ca^2+^-ATPases (SERCAs), in their slow-twitch type I skeletal muscle fibers to determine the effects on SERCA function. As expected with a 6- to 7-fold overexpression of phospholamban, SERCA dysfunction was evident in *Pln*^OE^ muscles, with marked reductions in rates of Ca^2+^ uptake, maximal ATPase activity and the apparent affinity of SERCA for Ca^2+^. However, our most significant discovery was that the soleus and gluteus minimus muscles from the *Pln*^OE^ mice displayed overt signs of myopathy: they histopathologically resembled human CNM, with centrally located nuclei, central aggregation of oxidative activity, type I fiber predominance and hypotrophy, progressive fibrosis and muscle weakness. This phenotype is associated with significant upregulation of muscle sarcolipin and dynamin 2, increased Ca^2+^-activated proteolysis, oxidative stress and protein nitrosylation. Moreover, in our assessment of muscle biopsies from three human CNM patients, we found a significant 53% reduction in SERCA activity and increases in both total and monomeric PLN content compared with five healthy subjects, thereby justifying future studies with more CNM patients. Altogether, our results suggest that the commercially available *Pln*^OE^ mouse phenotypically resembles human CNM and could be used as a model to test potential mechanisms and therapeutic strategies. To date, there is no cure for CNM and our results suggest that targeting SERCA function, which has already been shown to be an effective therapeutic target for murine muscular dystrophy and human cardiomyopathy, might represent a novel therapeutic strategy to combat CNM.

## Abstract (precis)

**Summary:** Phospholamban overexpression in mouse slow-twitch muscle impairs SERCA function and causes histopathological features associated with human centronuclear myopathy.

## INTRODUCTION

The sarco(endo)plasmic reticulum Ca^2+^-ATPase (SERCA) pumps catalyze the active transport of Ca^2+^ into the sarcoplasmic reticulum (SR) and play a crucial role in muscle relaxation and the maintenance of resting intracellular Ca^2+^ [Ca^2+^]_i_, which ranges from 30 to 100 nM in skeletal muscle (Tupling, 2009; Schiaffino and Reggiani, 2011). Phospholamban (PLN) is a small, 52-residue SR protein capable of physically interacting with and inhibiting SERCA activity by reducing its apparent affinity for Ca^2+^ (Morita et al., 2008). Mouse (Song et al., 2004) and rabbit (Pattison et al., 2008) models overexpressing PLN in slow-twitch (type I) muscle fibers (*Pln*^OE^) have been generated by attaching a *Pln* transgene to the β-myosin heavy chain (β-MHC) promoter, which preferentially directs high levels of expression of type I skeletal-muscle-specific transgenes (Rindt et al., 1993, 1995; Knotts et al., 1994). In the *Pln*^OE^ rabbit, obvious signs of muscular dystrophy, including central nucleation, severe muscle wasting, fibrosis, fatty infiltration and muscle weakness, were observed in soleus muscles, which are rich in type I fibers (Pattison et al., 2008). In contrast, *Pln*^OE^ mice exhibited soleus muscle atrophy, but no other signs of myopathy were visualized under the light microscope (Song et al., 2004).

We purchased these commercially available *Pln*^OE^ mice and their wild-type (WT) littermates (000067-MU, FVB/N background, Mutant Mouse Regional Resource Centre, Columbia, MO) to assess SERCA function because the effect of PLN overexpression on SERCA activity and Ca^2+^ transport in skeletal muscle remains uncharacterized. Unlike the study by Song et al. (2004), we discovered that the postural soleus and gluteus minimus muscles from *Pln*^OE^ mice exhibited overt signs of myopathy. Collectively, our data suggest that the *Pln*^OE^ mouse is a novel myopathy model resembling human centronuclear myopathy (CNM), with additional dystrophic features and potential core-like lesions.

## RESULTS

### SERCA function is impaired in *Pln*^OE^ muscle

**Fig. 1.**
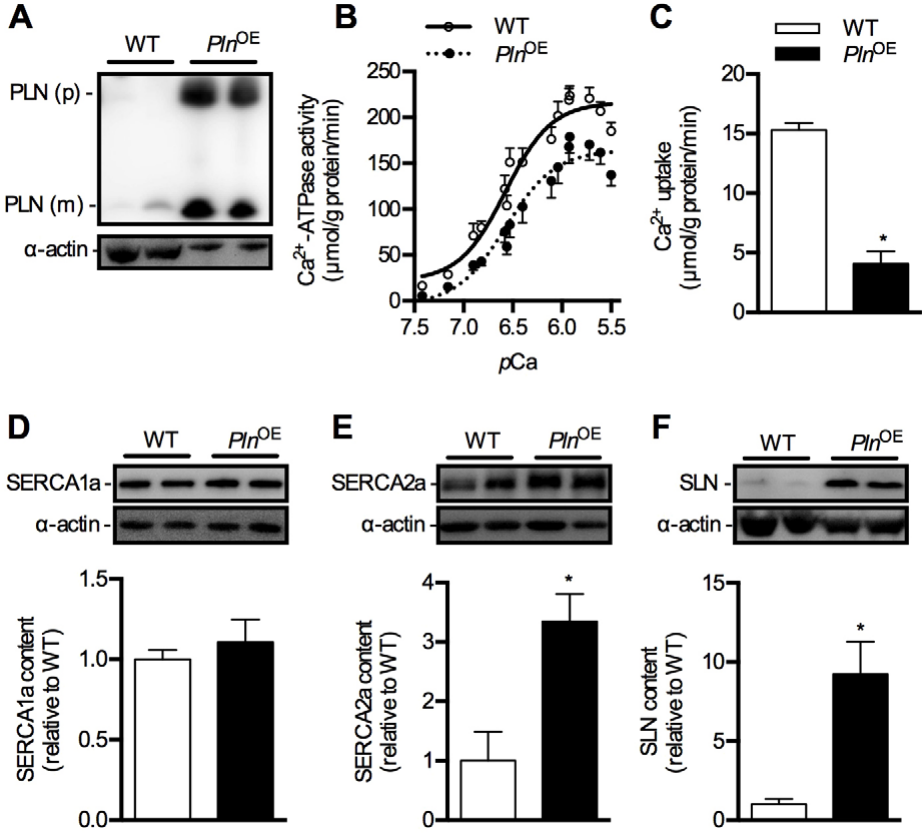
**SERCA function in soleus muscles in *Pln*^OE^ mice at 4-6 months of age.** (A) Western blotting for PLN in WT and *Pln*^OE^ mice from soleus muscle homogenates. For WT mice, 25 μg of total protein was loaded, whereas only 2.5 μg was required for *Pln*^OE^ mice to detect PLN protein. (B) Ca^2+^-ATPase activity–*p*Ca curves in WT (*n*=5) and *Pln*^OE^ mice (*n*=6) in the presence of the Ca^2+^ ionophore. (C) Ca^2+^ uptake assessed in soleus muscles from WT (*n*=4) and *Pln*^OE^ mice (*n*=5). (D-F) Western blotting for SERCA1a (D), SERCA2a (E) and SLN (F) in soleus muscle from WT and *Pln*^OE^ mice (*n*=6 per genotype). Actin was used as a loading control and all values are expressed relative to WT. *P≤0.05 versus WT. All values are presented as means±s.e. PLN (p), PLN pentamer; PLN (m), PLN monomer.

*Pln*^OE^ mice were generated in order to examine the functional importance of PLN in skeletal muscle; however, in the only study published using these mice (Song et al., 2004), no measurements of SERCA function were reported because of technical limitations. In order to better define the functional role of PLN in skeletal muscle we assessed SERCA function in these mice, focusing our analyses on the postural muscles – soleus and gluteus minimus – since these muscles normally contain approximately 55-65% and 27% type I fibers, respectively. However, these type I fiber levels increase in response to overexpression of PLN (discussed below). Western blotting of whole homogenates demonstrated clear overexpression of PLN in these muscles because only 2.5 μg of total protein was loaded for *Pln*^OE^ mice compared with the 25 μg required for WT mice (Fig. 1A and supplementary material Fig. S3A). Specifically, in the *Pln*^OE^ soleus relative to WT (*n*=7-9 per genotype), monomeric and pentameric PLN levels, normalized to actin, were 6.3-fold (*P=*0.00001) and 80-fold (*P=*0.0007) higher, respectively. In the *Pln*^OE^ gluteus minimus muscles relative to WT (*n*=5-6 per genotype), monomeric and pentameric PLN levels were 6.7-fold (*P=*0.0001) and 23-fold (*P=*0.0001) higher, respectively. In addition to the analysis in homogenates, we also performed single-fiber western blot analyses confirming overexpression of PLN specific to type I fibers in this model (supplementary material Fig. S1). In contrast, PLN was barely detectable in some WT type I and type IIA fibers (supplementary material Fig. S1). This corresponds well with the fact that PLN expression in rodent skeletal muscle is far lower than that in human skeletal muscle, where PLN can be found in all type I and type IIA fibers in the vastus lateralis (Fajardo et al., 2013).

**RESOURCE IMPACT**

**Background**

Phospholamban (PLN) is a well-known inhibitor of the sarco(endo)plasmic reticulum Ca^2+^-ATPase (SERCA) pumps in muscle that maintain low levels of cytosolic Ca^2+^ and that play a crucial role in muscle contraction. The importance of PLN regulation of SERCA function in cardiac muscle health and disease is well established but whether PLN plays a similar role in skeletal muscle disease remains unknown. Centronuclear myopathy (CNM) is a congenital myopathy characterized by muscle weakness, centrally located nuclei, type I fiber predominance and central aggregation of oxidative activity in the skeletal muscles. To date, there is no curative treatment for CNM and the exact mechanism leading to these skeletal muscle defects remains unknown. Thus, animal models that accurately recapitulate these histological abnormalities are required.

**Results**

In this study, the authors characterize a mouse model (*Pln*^OE^) in which PLN is overexpressed in type I skeletal muscle fibers. They show that SERCA function is greatly impaired in this model and that the soleus and gluteus minimus muscles, which normally contain many type I fibers, from *Pln*^OE^ mice exhibit phenotypes that resemble human CNM including centrally located nuclei, type I fiber predominance, central aggregation of oxidative activity and weakness. These muscles also present with progressive atrophy, fibrosis and potential core formations. Finally, the authors report that SERCA function was on average 53% lower in muscle biopsies from three patients with CNM compared with biopsies from five healthy individuals, whereas PLN expression seemed to be elevated in biopsies from patients with CNM compared with healthy controls.

**Implications and future directions**

These results identify a novel CNM mouse model that can be used for the investigation of the mechanisms underlying CNM and for the development of therapeutic strategies. The findings also suggest that studies to assess the role of PLN and SERCA dysfunction in human and animal CNM and myopathy in general might be worthwhile. To date, 25-30% of CNM cases remain genetically unresolved but several mutations in the human gene encoding PLN are known to lead to SERCA inhibition and cardiac complications. Given that many clinical cases of CNM present with cardiomyopathy, future studies should determine whether skeletal muscle defects are also present in patients harboring *PLN* mutations. Finally, these findings add to the notion that aberrant Ca^2+^ regulation is central to many cardiac and skeletal muscle diseases and that targeting SERCA function might represent a viable therapeutic strategy for these diseases.

In agreement with the known effects of PLN overexpression on SERCA function in HEK 293 cells and transgenic hearts (MacLennan and Kranias, 2003; Bhupathy et al., 2007), we observed a reduction in SERCA's apparent affinity for Ca^2+^, as indicated by a higher *K*_Ca_ value and a corresponding rightward shift in the activity–*p*Ca curve in soleus homogenates prepared from *Pln*^OE^ mice compared with WT (Table 1 and Fig. 1B). Furthermore, maximal rates of SERCA activity (−27%) and rates of Ca^2+^ uptake (−74%) were reduced in the *Pln*^OE^ soleus compared with WT (Fig. 1B and C and Table 1). The SERCA dysfunction in *Pln*^OE^ soleus cannot be explained by altered SERCA expression since western blot analysis showed that SERCA1a content is similar in WT and *Pln*^OE^ muscles (Fig. 1D), whereas a significant 3-fold increase in SERCA2a expression was observed in the soleus of *Pln*^OE^ mice (Fig. 1E). Sarcolipin (SLN), a small SR protein that is structurally and functionally homologous with PLN (Asahi et al., 2002, 2003), was also found to be upregulated 9-fold in the soleus of *Pln*^OE^ mice compared with WT (Fig. 1F). As SLN can inhibit SERCA pumps alone or in combination with PLN (Asahi et al., 2003), these results suggest that SLN may also contribute to the impaired SERCA function we observed in *Pln*^OE^ muscles. Indeed, the forced overexpression of SLN acutely in rat soleus reduces SR Ca^2+^ transport (−31%) and causes severe contractile dysfunction (Tupling et al., 2002).

**Table 1.**
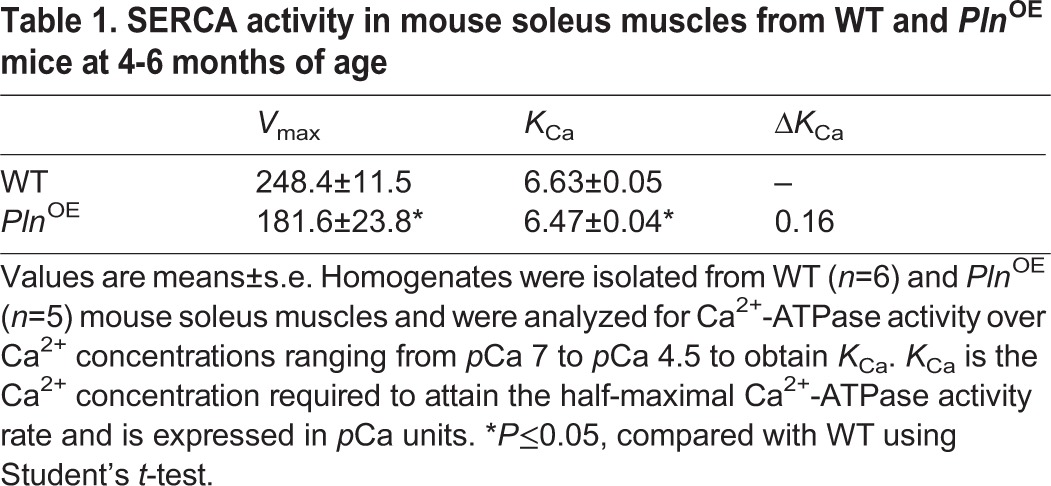
**SERCA activity in mouse soleus muscles from WT and *Pln*^OE^ mice at 4-6 months of age**

### Soleus muscle atrophy and Ca^2+^-activated proteolysis in *Pln*^OE^ mice

**Fig. 2.**
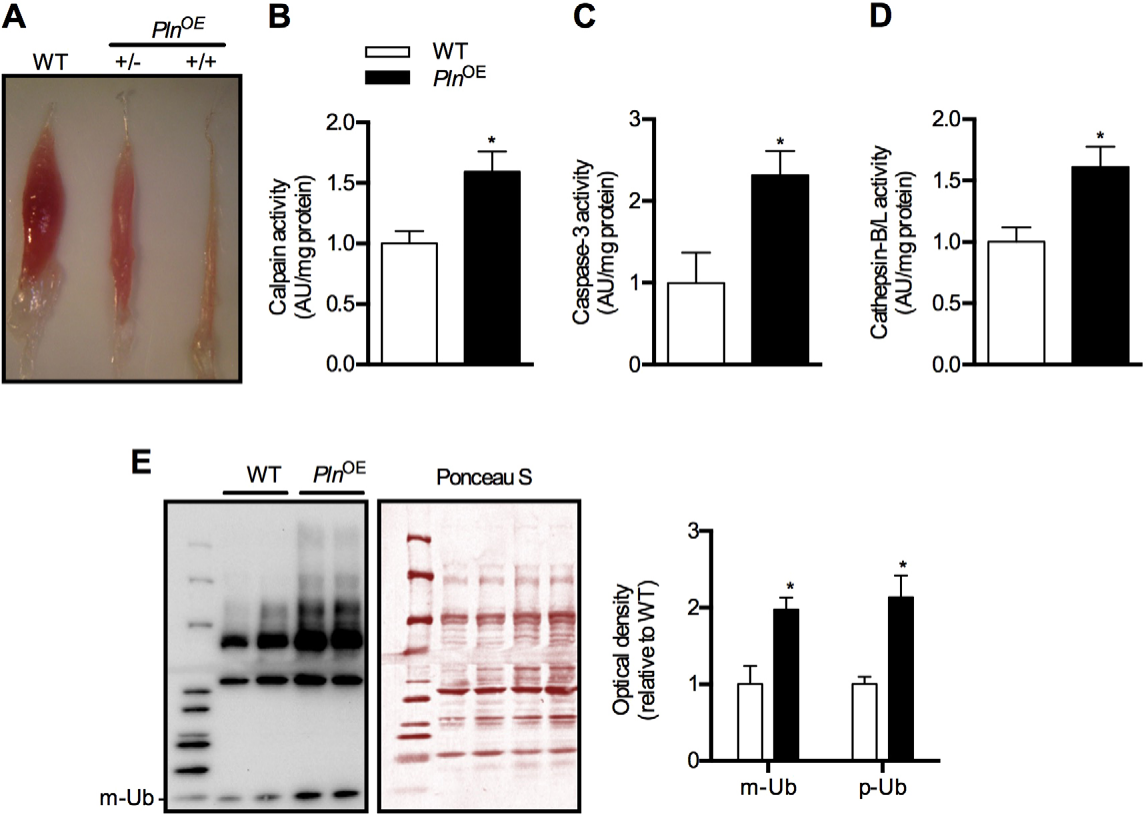
**Soleus muscles of *Pln*^OE^ mice are atrophied at 4-6 months of age.** (A) Representative images of soleus muscles extracted from WT and *Pln*^OE^ mice. +/−, heterozygous; +/+, homozygous. (B) Calpain activity in soleus homogenates from WT (*n*=4) and *Pln*^OE^ mice (*n*=5). (C) Caspase-3 activity in soleus muscle homogenates from WT (*n*=4) and *Pln*^OE^ mice (*n*=7). (D) Cathepsin-B/L activity in soleus homogenates from WT and *Pln*^OE^ mice (WT, *n*=4; *Pln*^OE^, *n*=6). Calpain activity, caspase-3 activity, and cathepsin-L activity are in arbitrary units normalized to mg protein and are presented relative to WT. (E) Total protein extracts from soleus muscles (15 μg) were immunoblotted with anti-ubiquitin (Ub) antibody. Ponceau stain was used as a loading control. The sum of optical densities from detectable ubiquitylated proteins (p-Ub) as well as the optical density of monomeric ubiquitin (m-Ub, 10 kDa) was measured and compared between genotypes (*n*=4 per group). Values were normalized to the sum of optical densities of bands visualized through Ponceau stain. *P≤0.05 versus WT. All values are presented as means±s.e.

Similar to previously described results (Song et al., 2004), we also observed atrophy of soleus muscles in *Pln*^OE^ mice (Fig. 2A and supplementary material Table S1). Interestingly, when breeding male and female *Pln*^OE^ mice, soleus muscles weighing ≤1 mg were observed, presumably from homozygous *Pln*^OE^ mice (+/+), because these muscles were no longer seen after breeding male *Pln*^OE^ mice with female WT mice (Fig. 2A). Owing to this limitation of the tissue, we restricted all of our analyses in the present study to heterozygous *Pln*^OE^ mice (+/−). In the study published by Song et al. (2004), a 35-40% reduction in soleus:body-weight ratio was observed in *Pln*^OE^ mice at 10-12 weeks of age. In our hands, assessment of soleus:body-weight ratio in *Pln*^OE^ mice at 1 month, 4-6 months and 10-12 months of age indicates a 21%, 43% and 41% reduction, respectively, when compared with WT mice (supplementary material Table S1). When we include age as a factor, absolute soleus weight and the soleus:body-weight ratios were significantly different at 4-6 months and 10-12 months, but not at 1 month. To elucidate the mechanisms behind the diminished soleus muscle mass, Song and colleagues reported an approximate 2-fold increase in calpain expression (Song et al., 2004). Calpains are a class of proteolytic enzymes that are activated by high levels of [Ca^2+^]_i_ (Murphy et al., 2006), and our results show that calpain activity was 1.5-fold higher in soleus from *Pln*^OE^ mice compared with WT (Fig. 2B), which probably results from reduced SERCA function and greater [Ca^2+^]_i_ in *Pln*^OE^ mice. We also observed a significant 1.5- to 2-fold increase in other proteolytic pathways, such as caspase-3, cathepsin-B/L activity and protein ubiquitylation (Fig. 2C-E), which suggests that the soleus muscle atrophy in *Pln*^OE^ mice is due to a general overall enhancement of proteolytic activity in those muscles.

### Central nuclei, progressive fibrosis and oxidative stress in *Pln*^OE^ mice

**Fig. 3.**
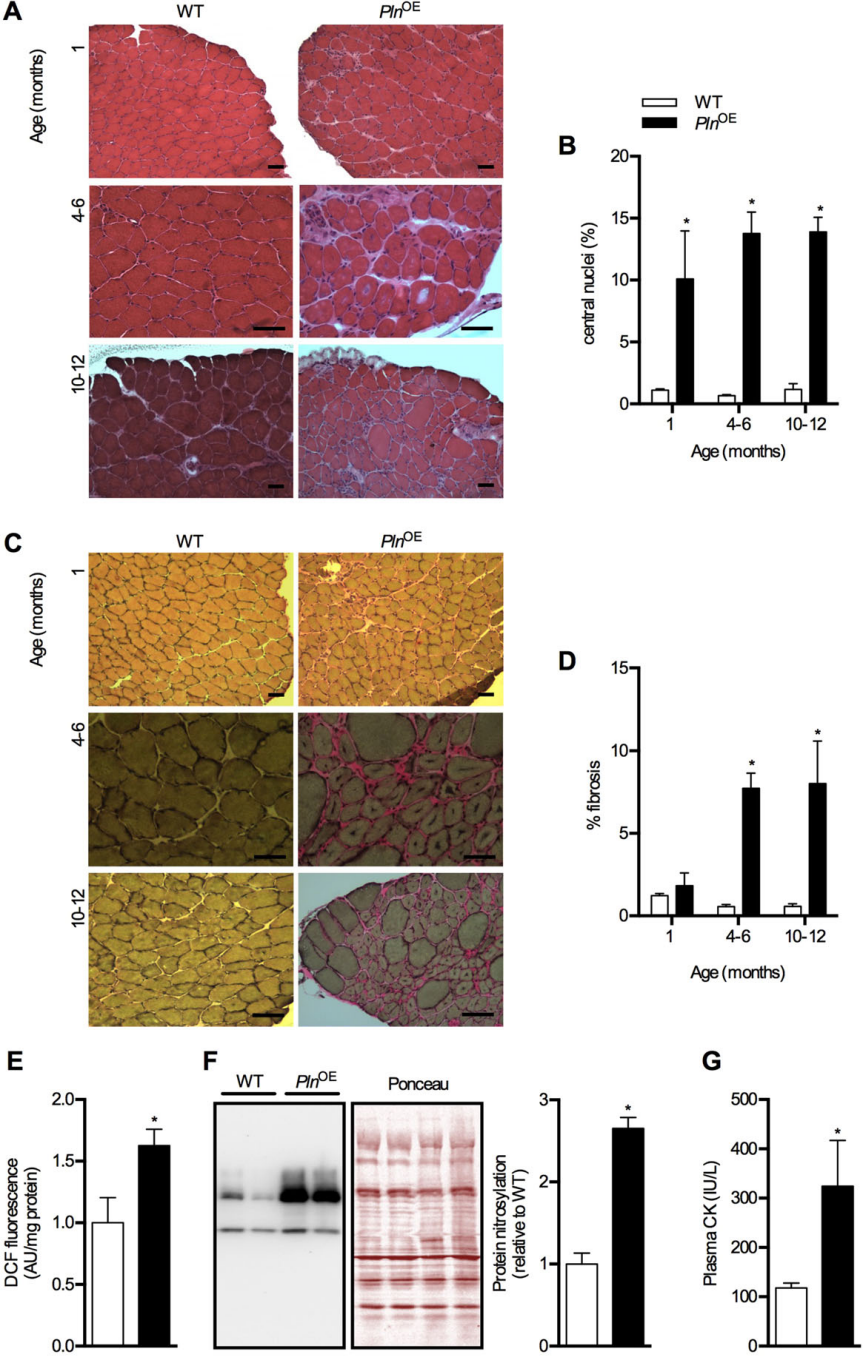
**Dystrophic features in soleus muscles from *Pln*^OE^ mice.** (A) H&E-stained sections of the soleus muscles from WT and *Pln*^OE^ mice at 1 month, 4-6 months and 10-12 months of age. (B) Percentage of fibers containing central nuclei in the soleus at 1 month, 4-6 months and 10-12 months of age (*n*=3 per genotype at each age with 300-600 fibers counted per mouse). (C) Van-Geison-stained sections of the soleus muscles from WT and *Pln*^OE^ mice at 1 month, 4-6 months and 10-12 months of age. (D) Quantification of fibrotic area in the soleus at 1 month, 4-6 months and 10-12 months of age (*n*=3-4 per group at each age). ImageJ software was used to quantify fibrotic area. (E) Reactive oxygen species generation in whole soleus determined using DCF assay at 4-6 months of age (WT, *n*=4; *Pln*^OE^, *n*=5). (F) Total protein extracts from soleus muscles (15 μg) were immunoblotted with anti-nitrotyrosine antibody. The sum of optical densities from detectable nitrosylated proteins (50-100 kDa) was measured and compared between genotypes at 4-6 months of age (*n*=6 per group). Values were normalized to the sum of optical densities of bands visualized through Ponceau stain. (G) Plasma CK levels in WT (*n*=5) and *Pln*^OE^ mice (*n*=9) at 4-6 months of age. Scale bars: 50 μm (A,C). *P≤0.05 versus WT using two-way ANOVA and Tukey's *post hoc* analysis for % central nuclei and % fibrosis; Student's *t*-test for DCF, protein nitrosylation and plasma CK. All values are presented as means±s.e.

As mentioned earlier, previous analyses of *Pln*^OE^ mice revealed no signs of myopathy under the light microscope (Song et al., 2004). Consequently, we were surprised that our histological analyses of the soleus muscles from *Pln*^OE^ mice showed obvious signs of pathology that were repeatedly observed (Fig. 3). In fact, the myopathy was at least partially consistent with the dystrophic-like phenotype observed in *Pln*^OE^ rabbits (Pattison et al., 2008), including greater central nucleation of fibers, fiber splitting, atrophy and progressive fibrosis, which became evident at 4-6 months of age (Fig. 3A-D). Corresponding well with the dystrophic phenotype, we also observed greater whole muscle oxidative stress, as indicated by DCF (2′,7′-dichlorofluorescein) fluorescence and protein nitrosylation – effects that may be explained by Ca^2+^ dysregulation (Altamirano et al., 2012). Furthermore, we found elevated plasma CK in 4- to 6-month-old *Pln*^OE^ mice (Fig. 3E-G); however, the relatively small increase in plasma CK suggests that the dystrophic-like phenotype in *Pln*^OE^ mice is a direct result of impaired SERCA activity and elevated [Ca^2+^]_i_, and is less likely to be caused by sarcolemmal damage per se. A similar finding has been reported in mice overexpressing transient receptor potential canonical 3, which led to greater Ca^2+^ influx, muscular dystrophy and only slightly elevated plasma CK as a result of a generally intact sarcolemmal membrane (Millay et al., 2009).

### CNM in the *Pln*^OE^ mouse

**Fig. 4.**
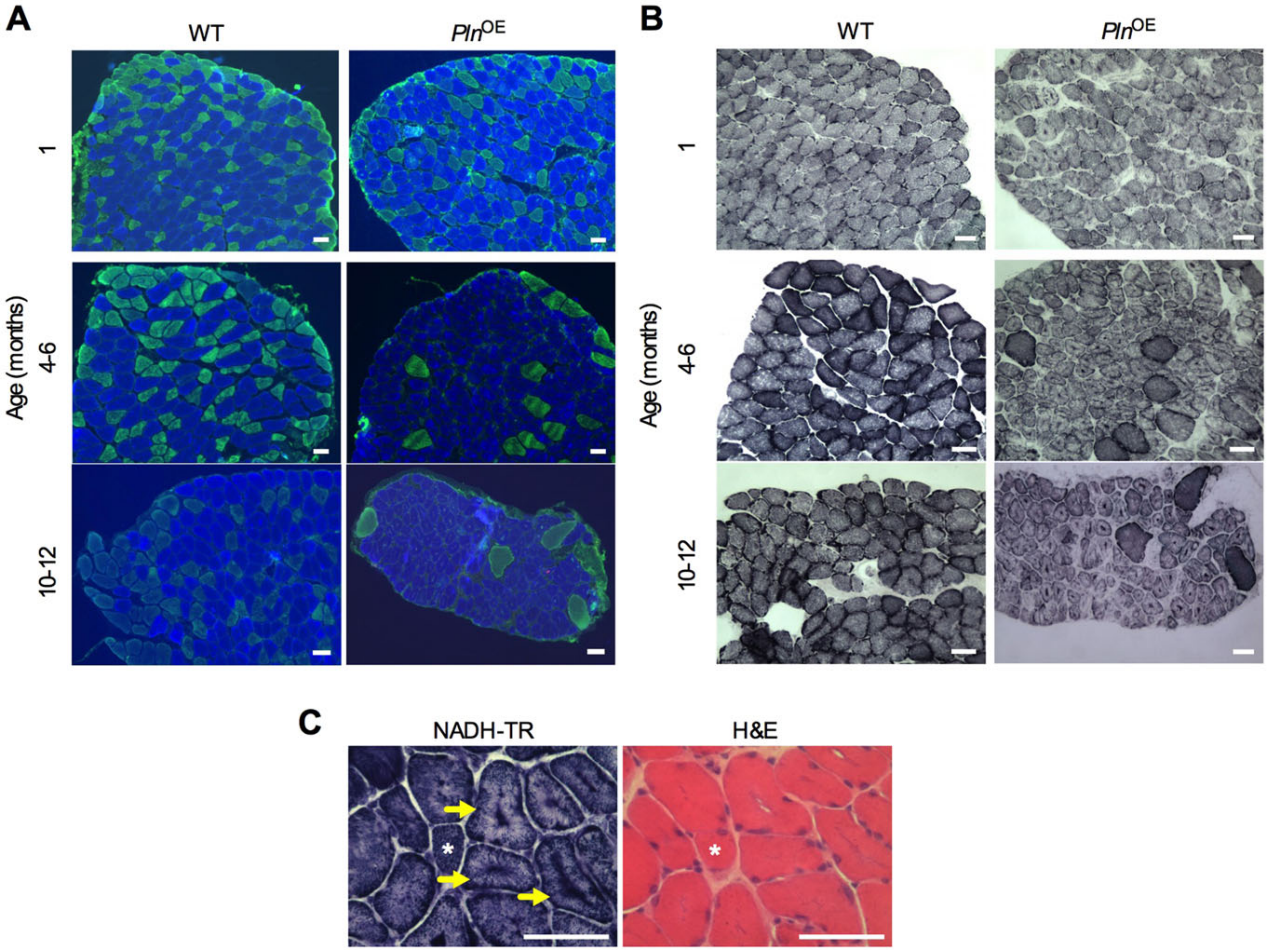
**Centronuclear myopathy in the soleus muscles from *Pln*^OE^ mice.** (A) Representative images of soleus muscles showing type I fiber predominance and hypotrophy. MHC immunofluorescence stained sections of the soleus muscles at 1 month, 4-6 months and 10-12 months of age. Cross sections were stained with antibodies against MHC to identify type I (blue), type IIA (green), type IIB (red) and type IIX (unstained) fibers. (B) Representative images of soleus muscles showing central accumulation of oxidative activity. Succinate dehydrogenase (SDH)-stained sections display central aggregation of oxidative activity in the *Pln*^OE^ mice at 1 month, 4-6 months and 10-12 months of age. (C) NADH-TR-stained cross sections demonstrating radiating SR strands in soleus muscles from *Pln*^OE^ mice (arrows). Asterisks represent the same fiber across cryosections. Scale bars: 50 μm.

Although central nucleation might be an active marker in muscle regeneration and a common feature in muscular dystrophy (Charge and Rudnicki, 2004; Dubowitz et al., 2013; Folker and Baylies, 2013), we observed only a small proportion of fibers that were positive for embryonic MHC (supplementary material Fig. S2), which is normally expressed transiently during muscle regeneration (Sartore et al., 1982; DiMario et al., 1991; Grady et al., 1997). These results, combined with the small elevation in plasma CK, suggest that very little degeneration–regeneration cycling is occurring in *Pln*^OE^ muscle. Therefore, the *Pln*^OE^ mouse does not closely resemble dystrophic myopathy; however, central nucleation in the face of low regeneration, progressive fibrosis, and moderate elevations in CK levels is also found in CNM (Jungbluth et al., 2008). CNM is a congenital myopathy that is diagnosed by the existence of three histopathological features found in muscle biopsies, which include: (1) central nucleation, (2) central aggregation of oxidative activity, and (3) predominance of type I fibers and hypotrophy (Jungbluth et al., 2008; Romero, 2010). Both central aggregation of oxidative activity and type I fiber hypotrophy were evident in soleus sections as early as 1 month of age in *Pln*^OE^ mice (Fig. 4A and B and Table 2). Type I fiber predominance was established at 4-6 months of age, whereas at 1 month, fibers were still transitioning towards the type I fiber distribution as the *Pln*^OE^ soleus had lower type IIA and greater type I/IIA content compared with WT (Fig. 4A and Table 2). The type I fiber predominance found from 4-6 months is in agreement with previous findings (Song et al., 2004), and the increase in the slow-twitch SERCA2a isoform in *Pln*^OE^ soleus (Fig. 1E) is also consistent with type I fiber predominance. Taken together, these results indicate that the *Pln*^OE^ mouse model is a phenocopy of the histological features found in human CNM. Importantly, the soleus muscle was not the only muscle affected by PLN overexpression because the postural gluteus minimus muscle, which normally comprises roughly 27% type I fibers in WT mice, also displayed signs of impaired SERCA function, CNM and endomysial fibrosis (supplementary material Fig. S3, Tables S2,S3). Despite a similar level of monomeric PLN overexpression, the relative impairment in SERCA function in the gluteus minimus muscles compared with the *Pln*^OE^ soleus was lower, with only a 20% reduction in the rates of Ca^2+^ uptake (supplementary material Fig. S3C). This might be explained by the fact that the gluteus minimus muscles have a greater number of type II fibers that do not overexpress PLN, which could mask the inhibition of SERCA pumps occurring in the type I fibers. In any event, both the *Pln*^OE^ soleus and gluteus minimus muscles resembled the histopathological features associated with CNM.

**Table 2.**
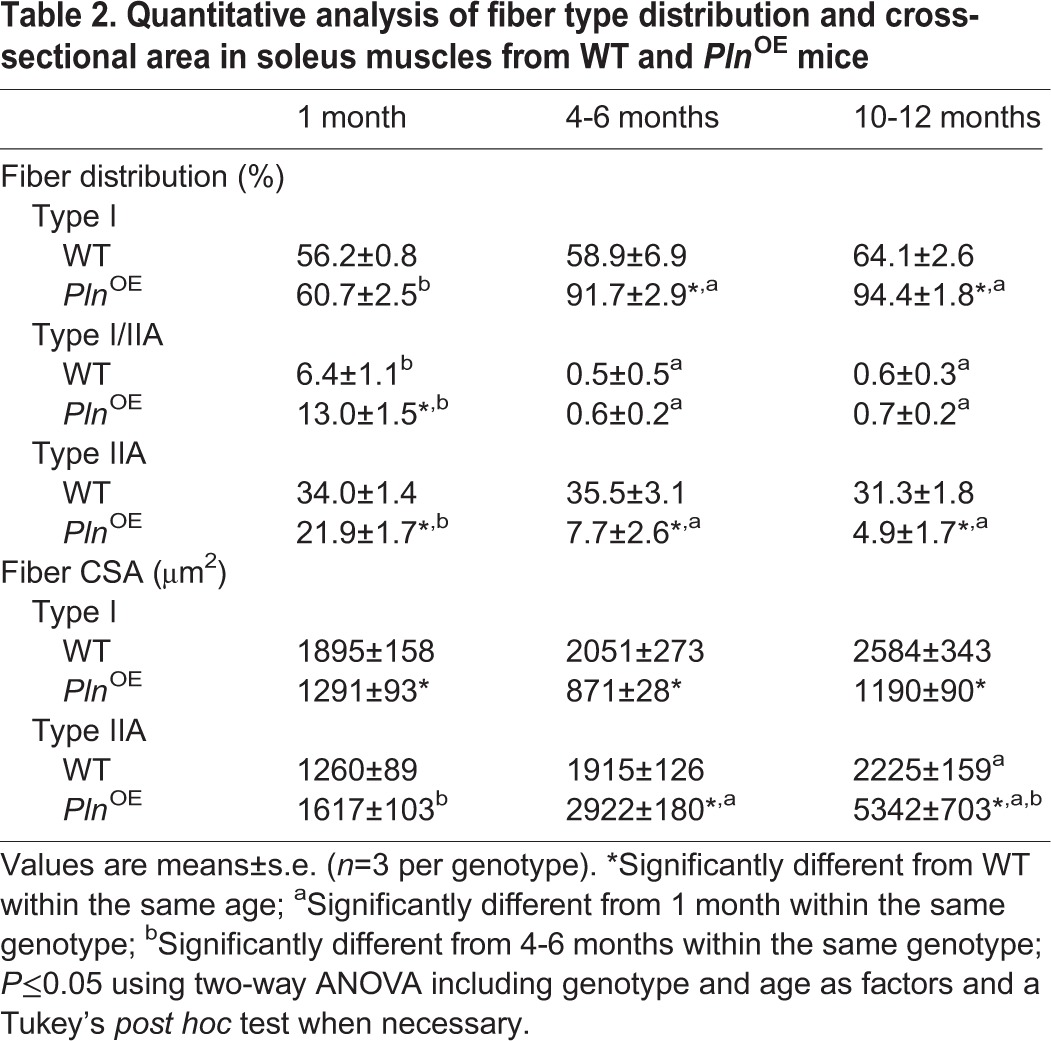
**Quantitative analysis of fiber type distribution and cross-sectional area in soleus muscles from WT and *Pln*^OE^ mice**

**Fig. 5.**
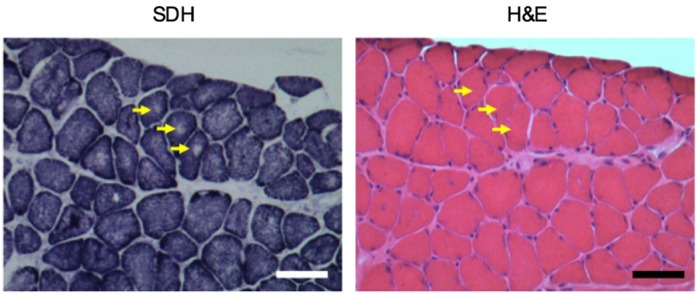
**Potential core-like lesions in *Pln*^OE^ mouse at 4-6 months of age.** SDH-stained sections from the soleus muscle show areas devoid of oxidative staining representing potential core-like lesions (yellow arrows). Corresponding H&E-stained serial section shows that the lack of oxidative staining with SDH is not due to the presence of a vacuole or an artifact in the muscle fiber.

To date, CNMs are known to be genetically heterogeneous (Dowling et al., 2014; Jungbluth and Gautel, 2014) and have been attributed to X-linked recessive mutations in the *MTM1* gene encoding myotubularin (Laporte et al., 1996), autosomal-dominant mutations in the *DNM2* gene encoding dynamin-2 (Bitoun et al., 2005) and the *BIN1* gene encoding amphiphysin-2 (Bohm et al., 2014), and autosomal-recessive mutations in *BIN1* (Nicot et al., 2007), *RYR1* encoding the SR ryanodine receptor (Jungbluth et al., 2007; Wilmshurst et al., 2010) and *TTN* encoding titin (Ceyhan-Birsoy et al., 2013). Many different structural abnormalities have been identified that aid in distinguishing between the various human CNMs (Romero, 2010; Jungbluth and Gautel, 2014). For example, the autosomal-dominant *DNM2*-related CNMs present with radiating SR strands and marked contrast of the diameters between type I and type II fibers (Romero, 2010). In addition to these features, increases in connective tissue and the presence of core formations can also be observed in *DNM2-*related CNMs (Jungbluth and Gautel, 2014). Since we observed radiating SR strands (Fig. 4C), type II fiber hypertrophy (Table 2 and supplementary material Table S3), progressive fibrosis (Fig. 3C and D) and potential core-like formations (Fig. 5) in the *Pln*^OE^ mice, our results suggest that the CNM phenotype found in these mice resembles autosomal-dominant *DNM2*-related CNM. However, because increases in endomysial connective tissue and the presence of core formations may also be observed in *RYR1*-related and *TTN*-related CNM (Jungbluth et al., 2007; Jungbluth and Gautel, 2014), the *Pln*^OE^ mouse also shares a resemblance to these forms of CNM. We use the term ‘potential core-like lesions’ because future studies with electron microscopy are required to verify the presence and characterize the structure of cores in the *Pln*^OE^ mouse.

### Muscle function in the *Pln*^OE^ mouse

General muscle weakness is common in cases of CNM and our analyses of contractile function in isolated intact soleus muscle, as well as whole-body endurance during treadmill exercise, demonstrate that muscle function is impaired in *Pln*^OE^ mice (supplementary material Fig. S4A-E). Specifically, we found that *Pln*^OE^ mice generate lower normalized force at both submaximal and maximal stimulation frequencies (30-100 Hz) (supplementary material Fig. S4D) and reached exhaustion much sooner than WT mice during a treadmill exercise test (supplementary material Fig. S4E). In agreement with previous findings (Song et al., 2004), we found that normalized twitch force generation of soleus muscle was not different between *Pln*^OE^ and WT mice, whereas the twitch kinetics were significantly slower in *Pln*^OE^ compared with WT (supplementary material Fig. S4A-C).

### PLN and SERCA dysfunction in human CNM

Thus far, it is evident that PLN overexpression in mouse skeletal muscle leads to CNM; however, whether PLN plays a primary pathogenic role in CNM remains unknown. In support of this notion, PLN protein was found to be upregulated in the tibialis anterior muscles from mice lacking microRNA 133a-1 and 133a-2, where CNM was observed (Liu et al., 2011). In addition, we obtained muscle biopsies from three patients with CNM and when compared with biopsies from five healthy controls, we found that the maximal rate of SERCA activity was significantly reduced by 53% (supplementary material Fig. S5A) and that monomeric PLN (*P=*0.12) and total PLN (*P=*0.08) content were greater (supplementary material Fig. S5B). Therefore, we provide the first indication of a potential role for SERCA dysfunction and elevated PLN in human CNM; however, caution should be taken when interpreting these results. Recently, we established through single-fiber western blotting and immunohistochemical analyses that PLN expression follows SERCA2a and MHC I expression, whereas SLN follows SERCA1a and MHC II expression in healthy human vastus lateralis (Fajardo et al., 2013). Since we observed a trend for higher MHC I (supplementary material Fig. S5C, *P=*0.10) and SERCA2a (supplementary material Fig. S5D, *P=*0.10) expression, and significant reductions in both SERCA1a (supplementary material Fig. S5E) and SLN (supplementary material Fig. S5F) in CNM muscles compared with healthy controls, it is possible that these differences, including upregulated PLN, are due mostly to the expected type I fiber predominance in CNM patients. Furthermore, in addition to the low sample size used in the present study, the CNM cases obtained were of notable age variability and were genetically unrelated, with one patient harboring a *DNM2* mutation (R369W) and the other two patients remaining genetically unresolved. Importantly, despite these limitations, we were still able to show a 53% reduction in SERCA activity and the expression patterns of the SERCA isoforms, SLN and PLN are consistent with the expected type I fiber predominance often found in CNM.

### Dynamin 2 levels in CNM

As a result of the dominant inheritance of the *DNM2* mutation, one theory is that *DNM2-*related CNM may be caused by a toxic gain-of-function in dynamin 2 (Cowling et al., 2014; Dowling et al., 2014). In support of this, overexpression of WT dynamin 2 in skeletal muscle results in features associated with CNM (Cowling et al., 2011; Liu et al., 2011). In addition, upregulation of dynamin 2 was observed in skeletal muscles from *mtm1*^–/y^ mice, which accurately models human X-linked myotubular myopathy (XLCNM), and heterozygous knockdown of dynamin 2 in the *mtm1*^–/y^ mouse improves muscle pathology and function (Cowling et al., 2014). We analyzed dynamin 2 protein content in *Pln*^OE^ muscles and found a 5-fold (supplementary material Fig. S6A) and 3-fold (supplementary material Fig. S6B) higher level in the soleus and gluteus minimus muscles, respectively, compared with WT. Whether dynamin 2 plays a pathological role in the CNM found in the *Pln*^OE^ mouse and the exact mechanism leading to its augmented expression requires further investigation.

Elevated levels of dynamin 2 are thought to also play a pathological role in human CNM because results from Laporte's laboratory have shown an approximate 1.5-fold increase in dynamin 2 expression in muscle lysates from three XLCNM patients compared with two healthy controls (Liu et al., 2011; Cowling et al., 2014). To confirm whether elevated dynamin 2 contributes to uman CNM pathology, we assessed the dynamin 2 protein content in the three CNM muscle biopsies obtained here; however, in contrast to what was previously found in human CNM (Liu et al., 2011; Cowling et al., 2014), we found a 35% reduction in dynamin 2 compared with healthy subjects (supplementary material Fig. S6C). Notwithstanding the aforementioned sample size and other limitations with our human CNM cases, these findings may suggest that reducing dynamin 2 as a therapeutic strategy may not be appropriate for all forms of human CNM as previously suggested (Demonbreun and McNally, 2014) and may be limited to XLCNM.

## DISCUSSION

To date, most studies concerning the physiological significance of PLN inhibition of SERCA have focused solely on its role in cardiac muscle health and disease (for a review, see MacLennan and Kranias, 2003). To our knowledge, our current study is the first to demonstrate an important role for PLN and SERCA dysfunction in skeletal muscle health and CNM pathology. Specifically, we obtained commercially available *Pln*^OE^ mice to determine PLN's effect on SERCA function in skeletal muscle and uncovered a mouse model that phenotypically resembles the histopathological features associated with human CNM. In addition, our results with three human CNM patients suggest that SERCA dysfunction, possibly through increased PLN expression, contributes to CNM pathology. Although it is possible that the predominance of type I fibers in human CNM may explain the elevations in PLN expression, it is important to consider our results from the *Pln*^OE^ mouse, which indicate that overexpression of PLN specifically in type I fibers can cause type I fiber predominance and CNM. Thus, future studies with more CNM patients that are similar in age and genetically related are required to better investigate whether PLN plays a primary pathological role in human CNM.

We cannot fully explain why we repeatedly observed myopathy in the *Pln*^OE^ mouse whereas Song and co-workers did not report such a finding (Song et al., 2004). Comparisons are difficult to make because SERCA function was not assessed and histology results were not shown in their study. Strain differences seem unlikely to account for the discrepant results since both transgenic lines were generated on an FVB/N background; however, it is possible that genetic drift and the level of PLN overexpression may be significant factors. Nevertheless, several results are in agreement between the two studies, including type I fiber predominance, atrophy, increased proteolytic markers and slower force kinetics in soleus muscle.

It has been estimated that 25-30% of human CNM cases remain genetically unresolved (Romero, 2010; Dowling et al., 2014) and our results raise the possibility that mutations in *Pln* leading to increased SERCA inhibition could be involved in those cases. Although *Pln* mutations either resulting in elevated PLN expression (Minamisawa et al., 2003) or reduced PLN phosphorylation (Schmitt et al., 2003; Haghighi et al., 2006) have been causally linked to human cardiomyopathy, it is unknown whether these mutations can affect skeletal muscle health. For example, deletion of arginine 14 (R14del) in the coding region of human *PLN* results in increased SERCA inhibition through increased monomeric PLN and reduced PLN phosphorylation at Ser16 by PKA (Haghighi et al., 2006). Interestingly, one patient with an R14del mutation in *PLN* developed late-onset mild dilated cardiomyopathy, but was initially evaluated in a muscle dystrophy clinic for a 25-year history of slowly progressive muscle weakness (DeWitt et al., 2006). Since there were no significant abnormalities in the staining patterns of sarcolemmal proteins, muscular dystrophy was discounted; however, to our knowledge, the existence of CNM was not tested. Recent reports of human cases of CNM coexisting with cardiomyopathy (Ceyhan-Birsoy et al., 2013; Agrawal et al., 2014; Gal et al., 2015) further highlight the importance of assessing the existence of CNM in skeletal muscles from patients with cardiomyopathy arising from *PLN* mutations.

Currently, the mechanisms leading to CNM are not completely understood; however, several mechanisms have been suggested, including abnormalities of triad structure and function (Al-Qusairi et al., 2009; Al-Qusairi and Laporte, 2011; Bohm et al., 2014; Dowling et al., 2014; Gibbs et al., 2014). As a result of these triad defects, aberrant Ca^2+^ handling and excitation–contraction (EC) coupling are also thought to be important for CNM pathogenesis. Indeed, the identification of mutations in *RYR1* leading to CNM (Jungbluth et al., 2007; Wilmshurst et al., 2010) further supports the notion that triad dysfunction, Ca^2+^ dysregulation and EC coupling are key pathogenic drivers of CNM (Dowling et al., 2014). Since the SERCA pumps regulate SR Ca^2+^ load and, thus, contractility, the SERCA pumps are also crucial for Ca^2+^ regulation and EC coupling. Thus, our results with the *Pln*^OE^ mice add further support to the hypothesis that defects in triad function, Ca^2+^ handling and EC coupling are important for CNM pathogenesis and extend the hypothesis by adding SERCA dysfunction as a potential pathogenic mechanism. Since abnormalities in triad structure are often found in human and animal CNM, future studies using electron microscopy should examine the triad structure in the *Pln*^OE^ mouse to determine whether structural abnormalities also contribute to the Ca^2+^ dysregulation seen in these mice. Furthermore, although areas devoid of oxidative (SDH) staining that cannot be explained by artifacts or the presence of vacuoles may represent core formations in the *Pln*^OE^ mouse, future studies with electron microscopy will confirm and characterize these potential core-like lesions.

One other interesting question raised by our study pertains to the role of SLN in CNM. Similar to PLN, SLN is a SERCA pump inhibitor (Asahi et al., 2002, 2003; Gorski et al., 2013), which suggests that the 7- to 9-fold upregulation in SLN protein found in the *Pln*^OE^ soleus and gluteus minimus muscles may contribute to the SERCA dysfunction, Ca^2+^ dysregulation and CNM pathology. Interestingly, this response in SLN expression is consistent with other mouse models of myopathy (Nakagawa et al., 2005; Ottenheijm et al., 2008; Al-Qusairi et al., 2009; Liu et al., 2011; Calvo et al., 2012; Schneider et al., 2013), but the role of SLN remains unknown. Our finding that SLN content was reduced in human CNM reveals a potential species difference and suggests that this SERCA regulator may have minimal contribution to the SERCA dysfunction and overall CNM pathology. To test whether or not SLN actively contributes to the CNM phenotype found in the *Pln*^OE^ mice, future studies targeting the *Sln* gene are required. Furthermore, a novel micropeptide, myoregulin (MLN), was found to be structurally and functionally homologous with both PLN and SLN (Anderson et al., 2015). When new antibodies targeting MLN protein expression become available, it will be interesting to determine its response and potential involvement in murine and human CNM and myopathy in general.

In summary, the commercially available *Pln*^OE^ mouse histopathologically resembles human CNM. To date, there is no cure for CNM and many unresolved questions remain, including the mechanisms leading to skeletal muscle defects in patients. Mechanistically, our results from the *Pln*^OE^ mouse are consistent with the hypothesis that triad dysfunction, aberrant Ca^2+^ handling and EC coupling are important for CNM pathogenesis; but whether it is higher cytosolic Ca^2+^ and/or altered Ca^2+^ dynamics that lead to CNM histopathology, and how the muscle translates this dysregulated Ca^2+^ into the CNM phenotype remains unknown and future studies using the *Pln*^OE^ mouse will prove valuable. Furthermore, treatment strategies aimed at improving SERCA function have already shown promise in murine muscular dystrophy (Goonasekera et al., 2011; Gehrig et al., 2012; Mazala et al., 2015) and human cardiomyopathy (Horowitz et al., 2011; Jessup et al., 2011; Zsebo et al., 2014). Thus, future studies that improve SERCA function through various modes, including alterations in PLN content and/or inhibitory function, in the *Pln*^OE^ mouse and other animal models of CNM, could lead to the development of viable therapeutic strategies for CNM patients.

## MATERIALS AND METHODS

### *Pln*^OE^ mice

*Pln*^OE^ mice were resuscitated from cryopreserved embryos by the mmRRC (000067-MU, FVB/N background) to generate a breeding colony with WT FVB/N mice in our facility. The *Pln* transgene was attached to the β-MHC promoter so that these mice overexpress PLN in their slow-twitch type I skeletal muscle fibers. Animals were housed in an environmentally controlled room with a standard 12 h:12 h light:dark cycle and allowed access to food and water *ad libitum*. Experiments were performed on littermate heterozygous *Pln*^OE^ and WT males that were between the ages of 1 and 12 months. Reintroduction of homozygous *Pln*^OE^ mice was avoided by breeding heterozygous male *Pln*^OE^ mice with female WT mice. Analysis of 287 newborn mice showed that WT and heterozygous *Pln*^OE^ mice were born at the expected mendelian frequency with a ratio of 137:150. All animal procedures were reviewed and approved by the Animal Care Committee of the University of Waterloo and are consistent with the guidelines established by the Canadian Council on Animal Care.

### Human subjects

Five untrained university students were used as healthy controls. All subjects were fully informed of all experimental procedures and all associated risks before written consent was obtained. Written approval for the research was granted by the Human Research Ethics Committee at the University of Waterloo. Three patients with CNM from McMaster University Medical Centre were used in this study. One female (55 years old) patient had CNM due to a missense mutation in dynamin 2 (*DNM2,* 1105C→T, R369W) whereas the other two male CNM patients (17 and 44 years old) remain genetically unresolved. Of the two unresolved cases, one underwent 163-gene next-generation sequencing, which included *MTM1*, *DNM2*, *RYR1*, *CACNA1S* and *TTN*. The other unresolved patient was screened for *MTM1* and *DNM2*; however, he did not return to complete a screen for additional genes. Written approval for the use of material from previously consented and diagnosed patients was granted by the Chair of the Human Research Ethics Committee at McMaster University. All muscle tissue samples (∼100 mg) were obtained from the vastus lateralis using the needle biopsy technique under suction (Tarnopolsky et al., 2011). These samples were homogenized and then frozen at −80°C prior to being used for western blotting and SERCA activity assays.

### SERCA activity and Ca^2+^ uptake

Ca^2+^-dependent SERCA activity was assessed in homogenates prepared from mouse (WT and *Pln*^OE^) soleus and gluteus minimus muscles and human vastus lateralis muscles over Ca^2+^ concentrations ranging from *p*Ca 7.5 to 4.5 in the presence of the Ca^2+^ ionophore A23187 (Sigma C7522) using a spectrophotometric plate reader assay that has been described previously (Duhamel et al., 2007). SERCA activity–*p*Ca curves were generated with GraphPad Prism™ by non-linear regression curve fitting using an equation for a general cooperative model for substrate activation. Ca^2+^ uptake was measured in homogenates in the presence of the precipitating anion, oxalate, using the fluorescent dye Indo-1 and a spectrofluorometer equipped with dual-emission monochromators (Tupling and Green, 2002). Rates of Ca^2+^ uptake assessed at a free cytosolic Ca^2+^ concentration of 1500 nM are reported.

### Antibodies

Primary antibodies against SERCA2a (2A7-A1), PLN (2D12), dynamin 2 (PA5-19800) and RyR (MA3-925) were obtained from Pierce Antibodies. The primary antibody for SERCA1a (A52) was a kind gift from Dr David MacLennan (University of Toronto) (Zubrzycka-Gaarn et al., 1984). The primary antibody directed against SLN was generated by Lampire Biological Laboratories (Fajardo et al., 2013). Anti-ubiquitin (P4D1) and anti-nitrotyrosine (189542) antibodies were obtained from Cell Signaling Technology and Cayman Chemicals, respectively. The primary antibody against α-actin (A4700) was obtained from Sigma-Aldrich. The primary antibodies against MHCI (BA-F8), MHCIIa (SC-71), MHCIIb (BF-F3), embryonic MHC (BF-F6) (Schiaffino et al., 1989; Lucas et al., 2000) and dystrophin (3B7) (Nguyen thi et al., 1990) were obtained from Developmental Studies Hybridoma Bank. Secondary antibodies for western blotting, goat anti-mouse IgG (peroxidase conjugated) and goat anti-rabbit IgG (peroxidase conjugated) were obtained from Santa Cruz Biotechnology. Secondary antibodies for immunofluorescence staining, Alexa Fluor 350 anti-mouse IgG_2b_, Alexa Fluor 488 anti-mouse IgG_1_ and Alexa Fluor 555 anti-mouse IgM, were obtained from Molecular Probes.

### Western blot analysis

Single-fiber western blots were performed as previously described (Fajardo et al., 2013) to determine fiber-type specificity of PLN overexpression. Briefly, single fibers extracted from soleus muscles of WT and *Pln*^OE^ mice were placed into 1× solubilizing buffer. Solubilized proteins were then separated using Tricine-based SDS-PAGE (6-13% layered gel). Separated proteins were then transferred onto 0.2 μm polyvinylidene difluoride (PVDF) membranes. Membranes were cut at the 75 kDa band (Western C Precision Plus™, Bio-Rad, CA, USA) and were immunoprobed with PLN (<75 kDa strip) and with MHCI (>75 kDa strip). Following this, membranes were immunoprobed with horseradish-peroxidase-conjugated secondary antibodies and antigen–antibody complexes were detected by Luminata Forte™ (Millipore, MA, USA) for PLN, and ECL Western Blot Substrate (BioVision, MA, USA) for MHCI. After detection of MHCI, the membrane was stripped and re-probed with MHCIIa and antigen–antibody complexes were detected using ECL Western Blot Substrate.

Similarly, western blot analysis was performed to determine expression levels of SLN, PLN, SERCA isofroms, dynamin 2, and RyR as well as levels of protein ubiquitylation (Ub) and nitrosylation (Ny) in mouse (WT and *Pln*^OE^) and human CNM muscles after homogenates were placed into 1× solubilizing buffer. Solubilized proteins from tissue homogenates, were separated using Tricine based SDS-PAGE (13% total acrylamide for PLN and SLN) or a standard SDS-PAGE (7.5% total acrylamide for SERCA isoforms and dynamin 2 and a 7.5-15% layered gel for Ub and Ny). Separated proteins were then transferred onto 0.2 μm PVDF membranes (PLN, SERCAs, Ub, Ny, RyR) or nitrocellulose membranes (SLN). Membranes were then immunoprobed with their corresponding primary antibodies. Following this, membranes were immunoprobed with horseradish-peroxidase-conjugated secondary antibodies and signals were detected by SuperSignal West Femto™ substrate (Pierce, Thermo Fisher Scientific Inc.) for SLN; Luminata Forte™ for SERCA2a, PLN, Ub, Ny and RyR; and ECL Western Blot Substrate for SERCA1a and dynamin 2. Quantification of optical densities was performed using GeneTools (Syngene, MD, USA) and values were normalized to total protein or α-actin.

### Histological, histochemical and immunofluorescence staining

Soleus and gluteus minimus muscles from WT and *Pln*^OE^ mice were removed and embedded in OCT compound (Tissue-Tek), frozen in liquid nitrogen-cooled isopentane, stored at −80°C, and cut into 10-μm-thick cryosections with a cryostat (Thermo Electronic) maintained at −20°C. Histological staining included H&E and Van Gieson and histochemical staining included succinate dehydrogenase (SDH) and NADH-TR activity. Images were acquired with a brightfield Nikon microscope linked to a PixeLink digital camera and quantified with Image-Pro PLUS analysis and ImageJ software. Immunofluorescence analysis of MHC expression was previously described (McMillan and Quadrilatero, 2011; Bloemberg and Quadrilatero, 2012; Fajardo et al., 2013) and performed with primary antibodies against MHCI, MHCIIa and MHCIIb. Additional immunofluorescence analysis of embryonic MHC expression was conducted with primary antibodies against embryonic MHC along with primary antibodies against dystrophin. Slides were visualized with an Axio Observer Z1 fluorescent microscope equipped with standard red, green, blue filters, an AxioCam HRm camera, and AxioVision software (Carl Zeiss). Quantification of fibers and cross-sectional area (CSA) was performed using ImageJ software.

### Proteolytic activity and DCF assays

Calpain, caspase-3 and cathepsin-B/L activity were determined in soleus muscle homogenates using the substrates, Suc-LLVY-AMC (Enzo-Life Sciences), Ac-DEVD-AMC (Alexis Biochemicals) and z-FR-AFC (Enzo Life Sciences), respectively (McMillan and Quadrilatero, 2011; Bloemberg et al., 2014). These fluorogenic substrates are weakly fluorescent but yield highly fluorescent products following proteolytic cleavage by their respective proteases. Fluorescence was measured using a SPECTRAmax Gemini XS microplate spectrofluorometer (Molecular Devices, Sunnyvale, CA) with excitation and emission wavelengths: 360 nm and 440 nm, respectively for caspase-3; 380 nm and 460 nm, respectively for calpain; 400 nm and 505 nm, respectively for cathepsin. Calpain activity was taken as the difference in fluorescence from homogenate incubated with and without calpain inhibitor. Calpain, caspase-3 and cathepsin activities were normalized to total protein content and expressed as fluorescence intensity in arbitrary units per mg of protein.

Generation of reactive oxygen species in soleus muscle homogenates from WT and *Pln*^OE^ mice was determined as previously described (McMillan and Quadrilatero, 2011) using 2′,7′-dichlorodihydrofluorescein-diacetate (DCFH-DA, Invitrogen, Carlsbad, CA) at 37°C. Cellular esterases hydrolyze DCFH-DA to the non-fluorescent DCFH, which can then be oxidized by a variety of ROS to form highly fluorescent DCF. Fluorescence was measured using a SPECTRAmax Gemini XS microplate spectrofluorometer with excitation and emission wavelengths of 490 nm and 525 nm, respectively. Fluorescence intensity was normalized to total protein content and expressed as arbitrary units per mg protein.

### Plasma CK analysis

WT and *Pln*^OE^ mice were anesthetized using somnotol (0.65 mg/kg body weight) and blood from the left ventricle was drawn into a heparinized syringe. Blood was centrifuged at 5000 ***g*** for 8 min and the plasma was decanted and stored at −80°C until analysis. CK activity was measured using a kinetic fluorometric assay as previously described (Szasz et al., 1976).

### Electrical stimulation and muscle contractility measurements

Experiments were performed on adult (4-6 month) WT and *Pln*^OE^ mice. Mice were killed by cervical dislocation, and the intact soleus muscles were removed and placed into a bath with oxygenated Tyrode solution (95% O_2_, 5% CO_2_) containing 121 mM NaCl_2_, 5 mM KCl, 24 mM NaHCO_3_, 1.8 mM CaCl_2_, 0.4 mM NaH_2_PO_4_, 5.5 mM glucose, 0.1 mM EDTA and 0.5 mM MgCl_2_, pH 7.3 (Lännergren et al., 2000), and was maintained at 25°C. Muscles were situated between flanking platinum electrodes driven by a biphasic stimulator (Model 710B, Aurora Scientific) and electrically evoked muscle force was assessed across a range of stimulation frequencies from 1 to 100 Hz. Data were analyzed using Dynamic Muscle Control Data Acquisition software (Aurora Scientific). Specifically, peak isometric force amplitude (mN) and the maximal rates of force development (+d*F*/d*t*) and relaxation (−d*F*/d*t*) were determined during a twitch and across the range of stimulation frequencies. Peak isometric force was then normalized to muscle weight (mN/g).

### Treadmill exercise

WT and *Pln*^OE^ mice were assessed for muscle performance by running them to exhaustion on an enclosed motorized treadmill. All animals exercised at a running speed of 8 m/min for 10 min followed by 10 min at 16 m/min and then allowed to reach exhaustion at 24 m/min at a 5° incline.

### Statistics

All values are presented as means±s.e. Statistical significance was set to *P*≤0.05. Comparisons between WT and *Pln*^OE^ mice were performed using Students *t*-test; however, a two-way repeated-measures ANOVA was used for force–frequency analysis, and when age was included as a factor. *Post hoc* testing was done using Tukey's HSD. Comparisons between healthy controls and human CNM patients were performed using Student's *t*-test.

## Supplementary Material

Supplementary Material
