# Supplementary material for: Phospholamban overexpression in mice causes a centronuclear myopathy-like phenotype
Source: Dis Model Mech. 2015 Aug 1;8(8):999–1009. doi: 10.1242/dmm.020859 (PMC4527296; doi:10.1242/dmm.020859)
Supplement: Supplementary Material [file supp_8_8_999__index.html]

Supplementary Material 

# Phospholamban overexpression in mice causes a centronuclear myopathy-like phenotype

## DMM020859 Supplementary Material

- Supplementary Material
