# Supplementary material for: Phospholamban overexpression in mice causes a centronuclear myopathy-like phenotype
Source: Dis Model Mech. 2015 Aug 1;8(8):999–1009. doi: 10.1242/dmm.020859 (PMC4527296; doi:10.1242/dmm.020859)
Supplement: Supplementary Material [file supp_020859_DMM020859supp.pdf]

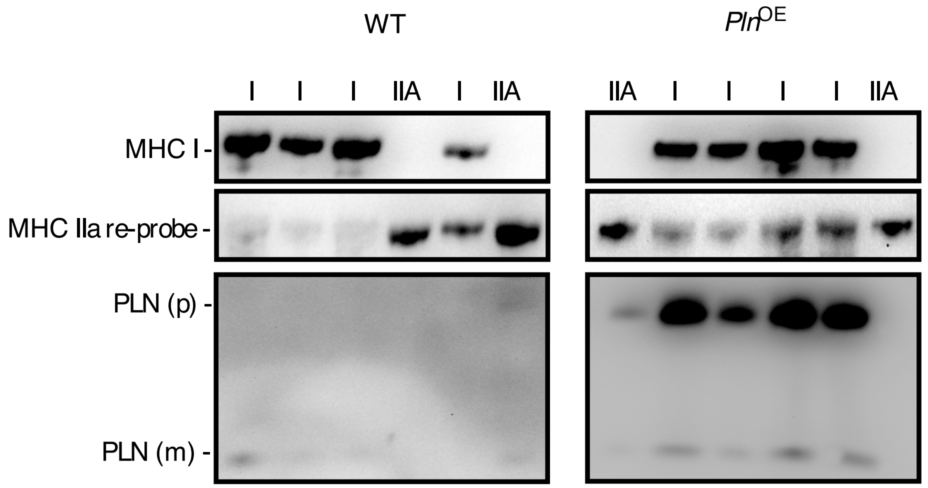

Fig. S1. PLN is overexpressed in type I fibres from soleus muscles. PLN expression in single fibres from WT mice was modestly detected in some type I and type IIA fibres and is clearly overexpressed in type I fibres from *Pln*^{OE} mice. MHCI was used to identify type I fibres. After detection of MHCI, membranes were stripped and re-probed with MHCIIa to identify type IIA fibres. Residual signals from MHCI were still present after the stripping and re-probing protocol. PLN (p), PLN pentamer; PLN (m), PLN monomer.

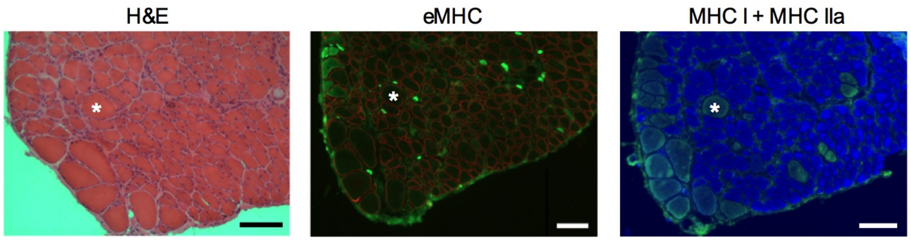

Fig. S2. Embryonic MHC in soleus muscles of *Pln*^{OE} mice**.** Centrally-located nuclei depicted through H&E staining cannot be fully explained by muscle regeneration as myosin heavy chain immunofluorescent stained sections of the soleus muscles show only minimal fibres positive for embryonic myosin heavy chain (eMHC, green). Cross sections were stained with dystrophin (red) to mark the sarcolemmal membrane of the fibre. Fibre-type analysis indicates that centrally-located nuclei are predominantly found in type I fibres (MHC I, blue) but can also be seen in some type IIA fibres (MHC IIa, green). Scale bars are set to 100 µm**.** White asterisks represent the same fibre in serial cross-sections.

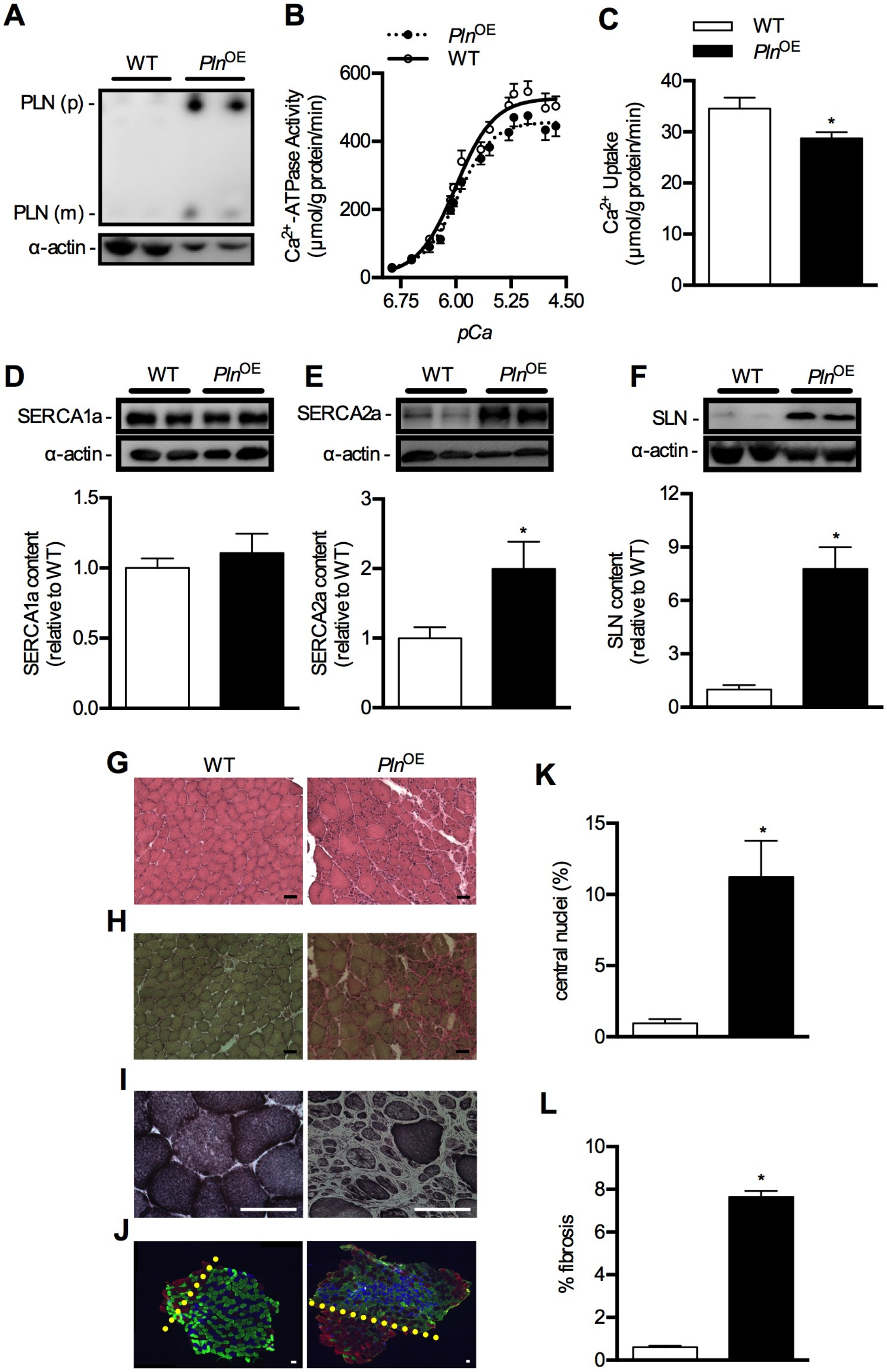
A
WT
Pln^OE
PLN (p) -
PLN (m) -
α-actin -
B
Pln^OE
WT
Ca2+-ATPase Activity (µmol/g protein/min)
600
400
200
0
6.75
6.00
5.25
4.50
pCa
C
WT
Pln^OE
Ca2+ Uptake (µmol/g protein/min)
40
30
20
10
0
*
D
WT
Pln^OE
SERCA1a -
α-actin -
SERCA1a content (relative to WT)
1.5
1.0
0.5
0.0
E
WT
Pln^OE
SERCA2a -
α-actin -
SERCA2a content (relative to WT)
3
2
1
0
*
F
WT
Pln^OE
SLN -
α-actin -
SLN content (relative to WT)
12
9
6
3
0
*
G
WT
Pln^OE
H
I
J
K
central nuclei (%)
15
10
5
0
*
L
% fibrosis
10
8
6
4
2
0
*

Fig. S3. Impaired SERCA function and CNM in gluteus minimus muscles from *Pln*^{OE} mice. (A) Western blotting for PLN in WT and *Pln*^{OE} mice from gluteus minimus homogenates. For WT mice, 25 µg of total protein was loaded whereas only 2.5 µg was required for *Pln*^{OE} mice to detect PLN protein. (B) Ca^{2+}-ATPase activity-*p*Ca curves in WT (n = 7) and *Pln*^{OE} mice (n = 6) in the presence of the Ca^{2+} ionophore . (C) Ca^{2+} uptake assessed in soleus and gluteus minimus muscles from WT (n = 6) and *Pln*^{OE} mice (n = 6). Western blotting for SERCA1a (D), SERCA2a (E), and sarcolipin (SLN; F) in soleus from WT and *Pln*^{OE} mice (n = 6 per genotype). Actin was used as a loading control and all values are expressed relative to WT. Representative gluteus minimus cross sections after H&E (G), Van Gieson (H), SDH (I), and immunofluorescent (J) staining. Cross sections were stained with MHC antibodies to identify type I (blue), type IIA (green), type IIB (red), and type IIX (unstained). Yellow dashed lines in (J) indicate the area that likely represents the gluteus medius and not the gluteus minimus. (K) Percent of fibres containing central nuclei in the gluteus minimus at 4-6 months (n = 4 per genotype at each age with 300-600 fibres counted per mouse). (L) Quantitation of fibrotic area in the soleus at 4-6 months of age (n = 4 per genotype). ImageJ software was used to quantify fibrotic area. *Significantly different from WT using Student's t-test, $P \leq 0.05$. All values are presented as mean ± standard error. Scale bars in *(G)*- *(J)* are set to 50 µm.

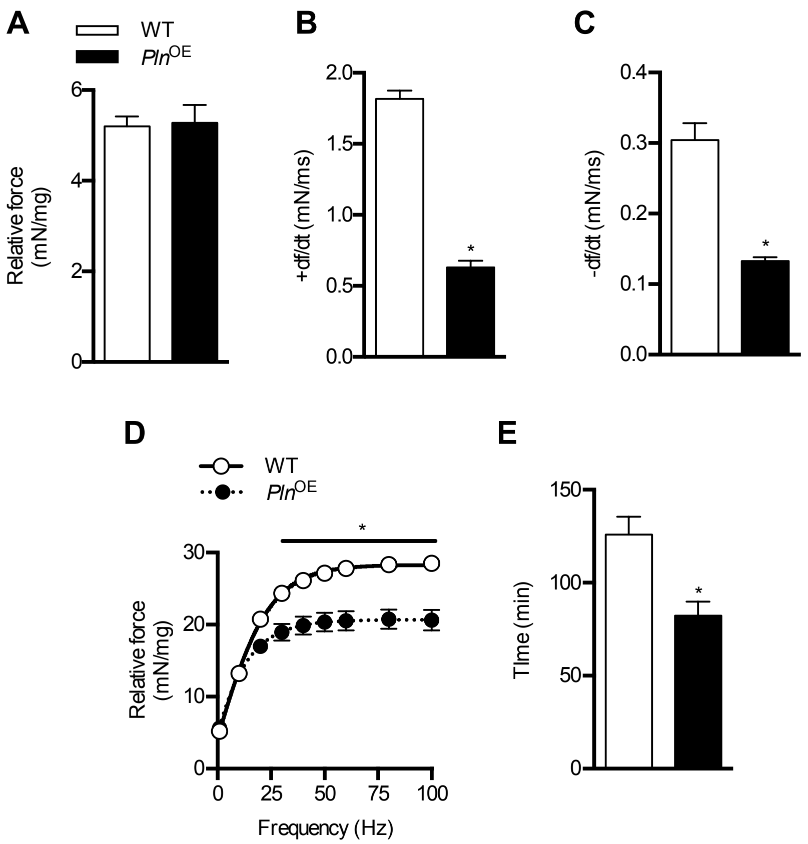

Fig. S4. Muscle function in *Pln*^{OE} mice. (A) Soleus isometric twitch force normalized to mass in 4-6 month old mice (WT n = 17, *Pln*^{OE} = 6). (B) Maximum rates of isometric twitch force development in soleus muscles from 4-6 month old mice (WT n = 17, *Pln*^{OE} = 6). (C) Maximum rates of isometric twitch force relaxation in soleus muscles from 4-6 month old mice (WT n = 17, *Pln*^{OE} = 6). (D) Force-frequency analysis in soleus muscles from 4-6 month old mice (WT n = 17, *Pln*^{OE} = 6). (E) Treadmill exercise performance of 4-6 month old mice measured as time (min) to exhaustion. * Significantly different from WT, $P \leq 0.05$. All values are presented as mean ± standard error.

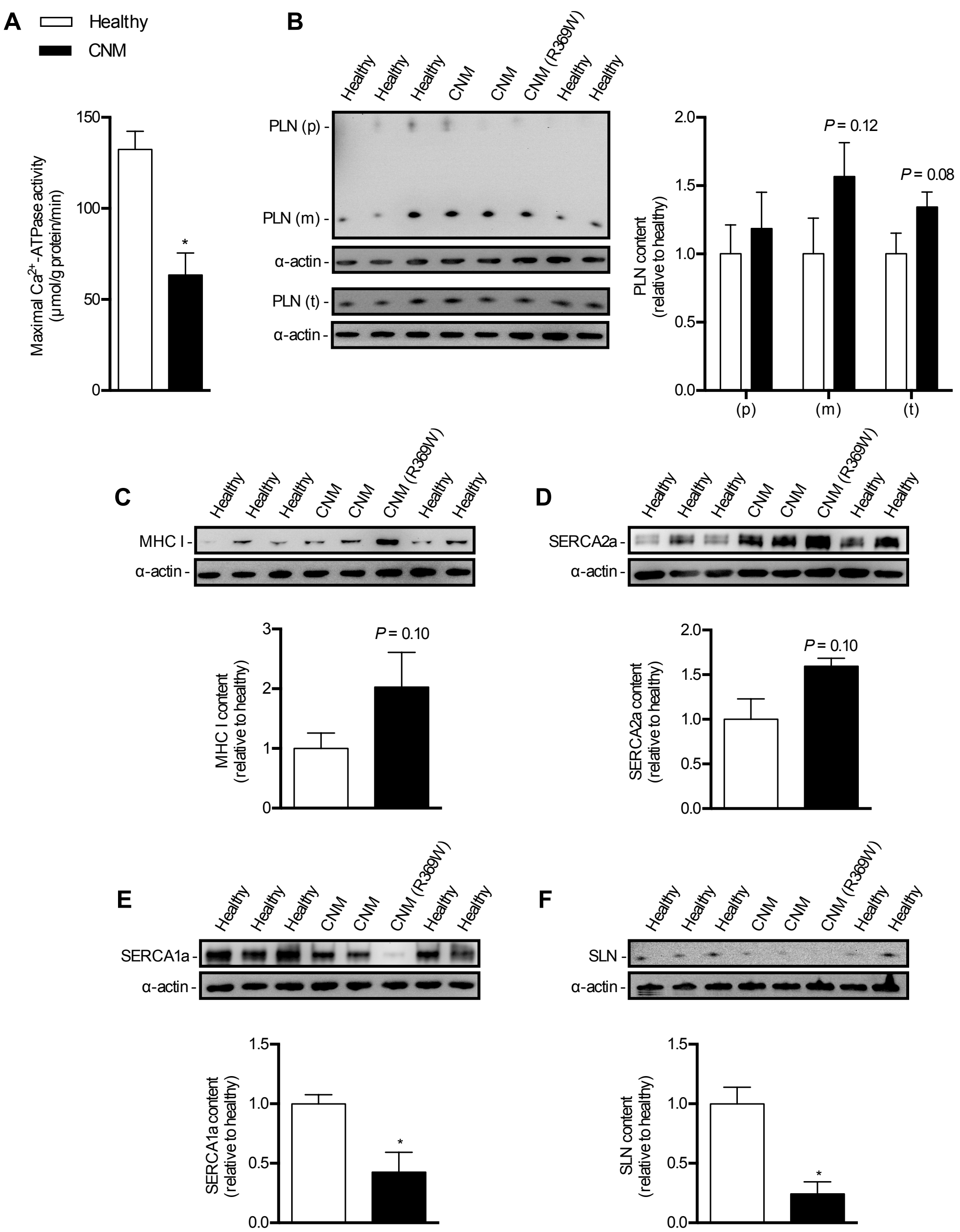

Fig. S5. Ca^{2+}-ATPase activity and protein expression in muscle biopsies from three patients with centronuclear myopathy (CNM). (A) Maximal rates of SERCA ATPase activity in biopsies from patients with CNM and healthy controls. Western blotting for PLN (B), MHC I (C), SERCA2a (D), SERCA1a (E), and SLN (F) in biopsies from patients with CNM and healthy controls. Actin was used as a loading control. *Significantly different from healthy using Student's t-test, $P \leq 0.05$. All values are presented as mean ± standard error. PLN (m), monomeric PLN; PLN (p) pentameric PLN; PLN (t), total boiled PLN. For PLN, MHCI, and SERCA2a 1 µg of total protein was loaded. For SERCA1a 0.5 µg of total protein was loaded. For SLN 2 µg of total protein was loaded. CNM (R369W), *DNM2*-CNM patient.

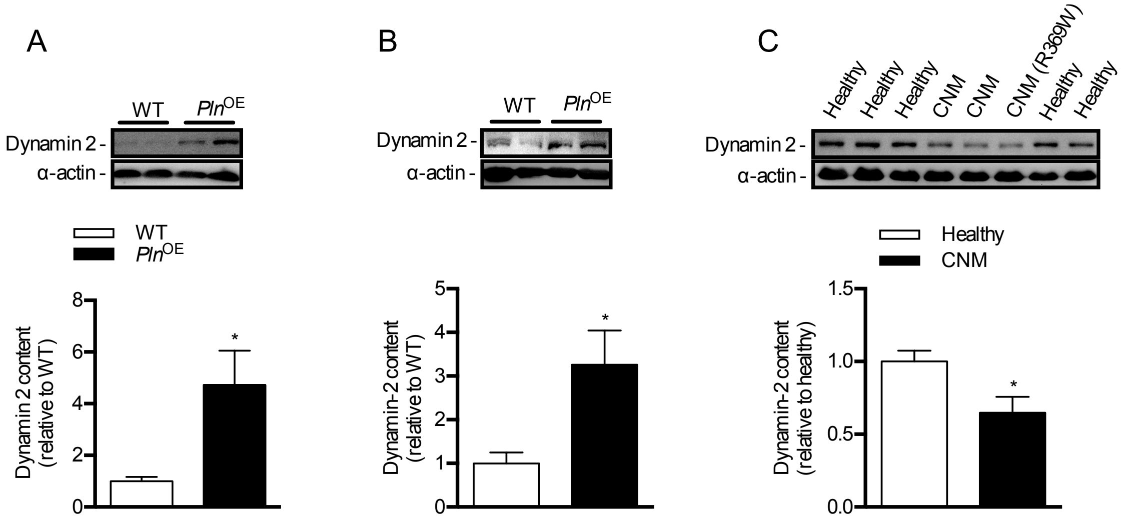

Fig. S6. Dynamin 2 protein content in the *Pln*^{OE} soleus (A) and *Pln*^{OE} gluteus minimus (B) muscles, and in muscle biopsies from three CNM patients compared to five healthy controls (C). For mouse soleus and gluteus minimus muscles, 7.5 µg of total protein were loaded, and 2 µg were loaded for human vastus lateralis. Actin was used as a loading control. *Significantly different from WT or healthy, using Student's t-test, $P \leq 0.05$. All values are presented as mean ± standard error. CNM (R369W), *DNM2*-CNM patient.

Table S1. Body weight, soleus weight, and soleus:body weight ratios in WT and *Pln*^{OE} mice at 1 month, 4-6 months, and 10-12 months of age.

	1 month	4-6 months	10-12 months
Body weight			
WT	22.9 ± 0.7	30.8 ± 0.8	33.1 ± 1.1
Pln^{OE}	22.4 ± 0.5	29.2 ± 0.5	33.7 ± 0.5
Soleus weight			
WT	4.5 ± 0.4	6.5 ± 0.4	5.8 ± 0.4
Pln^{OE}	3.3 ± 0.3	3.6 ± 0.3*	4.0 ± 0.1*
Soleus:body weight			
WT	0.19 ± 0.01	0.21 ± 0.01	0.17 ± 0.01
Pln^{OE}	0.15 ± 0.01	0.12 ± 0.01*	0.10 ± 0.01*

Values are means ± standard error. 1 month (WT, n = 8; *Pln*^{OE}, n = 8); 4-6 months (WT, n = 15; *Pln*^{OE}, n = 15); 10-12 months (WT, n = 8; *Pln*^{OE}, n = 6). *Significantly different from WT within the same age group, $P \leq 0.05$ using two-way ANOVA including genotype and age as factors and a Tukey's post-hoc test when necessary.

Table S2. SERCA activity in gluteus minimus muscles from WT and *Pln*^{OE} mice at 4-6 months of age.

Genotype	V_{max}	K_{Ca}	$\Delta p\text{Ca}_{50}$
WT	558.5 ± 29.1	5.99 ± 0.02	-
Pln^{OE}	477.1 ± 24.5*	5.96 ± 0.01	0.03

Values are means ± standard error. Homogenates were isolated from WT and *Pln*^{OE} mouse gluteus minimus muscles and were analyzed for Ca^{2+}-ATPase activity over Ca^{2+} concentrations ranging from *p*Ca 7 to *p*Ca 4.5 to obtain K_{Ca}. K_{Ca} is the Ca^{2+} concentration required to attain the half-maximal Ca^{2+}-ATPase activity rate and is expressed in *p*Ca units. *Significantly different from WT using Student's t-test, $P \leq 0.05$. A trend was observed for the K_{Ca} in the gluteus minimus muscles from WT and *Pln*^{OE} mice ($P = 0.10$).

Table S3. Quantitative analysis of fibre type distribution and cross-sectional area (CSA) in gluteus minimus muscles from WT and *Pln*^{OE} mice at 4-6 months of age.

	Fibre distribution (%)	Fibre CSA (μm^2)
Type I		
WT	26.9 ± 0.5	2713 ± 102
Pln^{OE}	47.7 ± 2.4*	1009 ± 180*
Type IIA		
WT	50.4 ± 1.7	2177 ± 110
Pln^{OE}	31.9 ± 2.9*	3026 ± 461
Type IIX		
WT	10.2 ± 1.0	2401 ± 118
Pln^{OE}	8.7 ± 0.7	3913 ± 406*
Type IIB		
WT	7.2 ± 0.7	4388 ± 189
Pln^{OE}	9.4 ± 0.3	5465 ± 267*

Values are means ± standard error (n = 5-6 per genotype). *Significantly different from WT within the same age; $P \leq 0.05$ using Student's t-test.
